# A Common Longitudinal Intensive Care Unit data Format (CLIF) to enable multi-institutional federated critical illness research

**DOI:** 10.1101/2024.09.04.24313058

**Published:** 2024-09-04

**Authors:** Juan C. Rojas, Patrick G. Lyons, Kaveri Chhikara, Vaishvik Chaudhari, Sivasubramanium V. Bhavani, Muna Nour, Kevin G. Buell, Kevin D. Smith, Catherine A. Gao, Saki Amagai, Chengsheng Mao, Yuan Luo, Anna K Barker, Mark Nuppnau, Haley Beck, Rachel Baccile, Michael Hermsen, Zewei Liao, Brenna Park-Egan, Kyle A Carey, XuanHan, Chad H Hochberg, Nicholas E Ingraham, William F Parker

**Affiliations:** 1 Division of Pulmonology, Critical Care, and Sleep Medicine, Rush University, Chicago, IL; 2 Department of Medicine, Oregon Health & Science University, Portland, OR; 3 Section of Pulmonary and Critical Care, Department of Medicine, University of Chicago, Chicago, IL; 4 Department of Medicine, Emory University, Atlanta, GA; 5 Division of Pulmonary and Critical Care, Department of Medicine, Northwestern University Feinberg School of Medicine, Chicago, IL; 6 Division of Health and Biomedical Informatics, Department of Preventive Medicine, Northwestern University Feinberg School of Medicine, Chicago, IL; 7 Division of Pulmonary and Critical Care, Department of Internal Medicine, University of Michigan, Ann Arbor, MI; 8 MacLean Center for Clinical Medical Ethics, University of Chicago Medicine, Chicago, IL; 9 Department of Medicine, University of Wisconsin School of Medicine and Public Health, Madison, WI; 10 Department of Medicine, University of Chicago, Chicago, IL; 11 Division of Pulmonary, Critical Care, and Sleep Medicine, Department of Medicine, Tufts University School of Medicine, Boston, MA; 12 Division of Pulmonary and Critical Care Medicine, Department of Medicine, Johns Hopkins University, Baltimore, MD; 13 Division of Pulmonary, Allergy, and Critical Care Medicine, Department of Medicine, University of Minnesota Medical School; University of Minnesota, Minneapolis, MN; 14 Department of Public Health Sciences, University of Chicago, Chicago, IL.

**Keywords:** Critical Care Data, Temperature Trajectory Modeling, Machine Learning

## Abstract

**Background:**

Critical illness, or acute organ failure requiring life support, threatens over five million American lives annually. Electronic health record (EHR) data are a source of granular information that could generate crucial insights into the nature and optimal treatment of critical illness. However, data management, security, and standardization are barriers to large-scale critical illness EHR studies.

**Methods:**

A consortium of critical care physicians and data scientists from eight US healthcare systems developed the Common Longitudinal Intensive Care Unit (ICU) data Format (CLIF), an open-source database format that harmonizes a minimum set of ICU Data Elements for use in critical illness research. We created a pipeline to process adult ICU EHR data at each site. After development and iteration, we conducted two proof-of-concept studies with a federated research architecture: 1) an external validation of an in-hospital mortality prediction model for critically ill patients and 2) an assessment of 72-hour temperature trajectories and their association with mechanical ventilation and in-hospital mortality using group-based trajectory models.

**Results:**

We converted longitudinal data from 94,356 critically ill patients treated in 2020–2021 (mean age 60.6 years [standard deviation 17.2], 30% Black, 7% Hispanic, 45% female) across 8 health systems and 33 hospitals into the CLIF format, The in-hospital mortality prediction model performed well in the health system where it was derived (0.81 AUC, 0.06 Brier score). Performance across CLIF consortium sites varied (AUCs: 0.74–0.83, Brier scores: 0.06–0.01), and demonstrated some degradation in predictive capability. Temperature trajectories were similar across health systems. Hypothermic and hyperthermic-slow-resolver patients consistently had the highest mortality.

**Conclusions:**

CLIF facilitates efficient, rigorous, and reproducible critical care research. Our federated case studies showcase CLIF’s potential for disease sub-phenotyping and clinical decision-support evaluation. Future applications include pragmatic EHR-based trials, target trial emulations, foundational multi-modal AI models of critical illness, and real-time critical care quality dashboards.

## INTRODUCTION

The intensive care unit (ICU) is an optimal setting for advanced clinical artificial intelligence (AI) projects because of voluminous longitudinal data and comprehensively captured outcome labels, demonstrated by exemplar deidentified electronic health record (EHR) databases such as MIMIC.^1–3^ AI applications in the ICU range from early warning systems for patient deterioration to programs designed to optimize resource allocation and personalize treatment recommendations.^4,5^ However, real-world ICU data science is often inefficient and difficult to scale because of challenges in acquiring, organizing, cleaning, and harmonizing EHR data. ICU EHR data are complex, highly temporally correlated, and subject to degradation through data capture and storage procedures designed for purposes other than research.^6^

Local EHR data repositories, or Electronic Data Warehouses (EDWs), are designed to maintain source data integrity and meet various institutional research and operational needs.^7^ EDWs often have unique idiosyncrasies, syntax, and data vocabularies which means extensive preprocessing is required before data can be analyzed for a specific use case.^8–11^ Established open-source common data models (CDMs), such as the Observational Medical Outcomes Partnership (OMOP),^12^ seek to address this data harmonization and standardization challenge for the entire EHR. However, the extract-transform-load (ETL) to a CDM is a major data engineering challenge, and data elements essential for the study of critical illness are not prioritized. Local CDM instances often completely omit granular critical illness data elements, such as ventilator settings for patients suffering from respiratory failure.^13,14^

To address these challenges and to support efficient and scalable data-driven critical care research, we developed the Common Longitudinal ICU Data Format (CLIF), a standardized data format for multi-center federated studies of critical illness. Recognizing that transforming raw data into analysis-ready structures is inherently domain-specific, we integrated the experiences and expertise of ICU clinician-scientists and data scientists, encoding this knowledge into a 1) clinically-driven entity-relationship model and a 2) minimum set of essential Common ICU Data Elements. Our overall objective was to create a robust critical illness research format that fully captures the dynamic clinical state of critical illness.

## METHODS

### CLIF Consortium Objectives and Process

We assembled a geographically diverse group of US-based physician-scientists and data scientists experienced in EHR-based clinical outcomes and AI research. Our guiding principles were: (1) efficient, clinically understandable data structures; (2) consistent and harmonizable data elements; (3) scalability and flexibility for future advancements; (4) federated analysis for collaborative research while maintaining data privacy and security; and (5) open-source development in line with the 2023 NIH Data Management and Sharing Policy and FAIR (Findable, Accessible, Interoperable, Reusable) data principles^15^.

We began weekly virtual meetings in July 2023 to develop operating procedures, terminologies, and quality control methods. We identified the practical challenges of using EHR data to study critical illness locally and across centers (Table 1) and addressed them when developing CLIF. CLIF is specifically designed for tasks like cohort discovery, temporal sequencing, and creating composite representations of clinical events, such as sepsis onset.

### CLIF entity-relationship model

**Figure 1.**
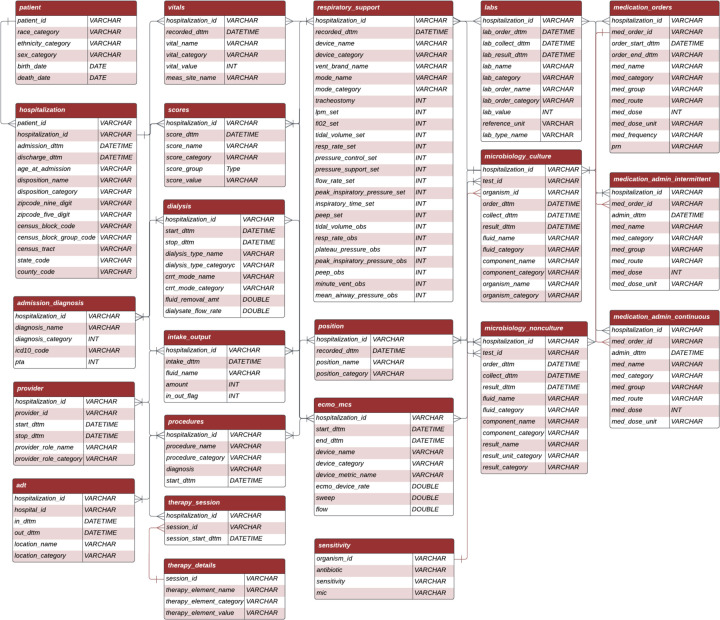
CLIF Entity Relationship Diagram. This diagram depicts the relationships between various tables in the Common Longitudinal ICU Data Format (CLIF) schema. The diagram includes the following 23 tables: 1. Patient 2. Hospitalization 3. Admission Diagnosis 4. Provider 5. ADT (Admission, Discharge, Transfer) 6. Vitals 7. Scores 8. Dialysis 9. Intake/Output 10. Procedures 11. Therapy session 12. Therapy Details 13. Respiratory Support 14. Position 15. ECMO (Extracorporeal Membrane Oxygenation) and Mechanical Circulatory Support (MCS) 16. Labs 17. Microbiology culture 18. Sensitivity 19. Microbiology non-culture 20. Respiratory Support 21. Medication Orders 22. Medication Admin Intermittent 23. Medication Admin Continuous Each table represents a specific aspect of ICU data, and lines between tables indicate how they are related through shared identifiers, primarily encounter_id. The depicted entity-relationship model was version 1.0, used for the case studies in this manuscript. The CLIF format is maintained with the git version control system, release 2.0.0 is available at https://clif-consortium.github.io/website/

CLIF’s entity-relationship (ER) model aligns with how critical care researchers organize and analyze clinical data in practice (Figure 1). It organizes various clinical data into 23 clinically relevant longitudinal tables linked by patient and hospitalization. These tables are organized by clinical information type and organ systems. CLIF’s ER model features specialized critical care tables such as respiratory support, continuous medications, dialysis, extra-corporeal membrane oxygenation/mechanical circulatory support, position (designed to identify prone mechanical ventilation), and scores (containing important clinical assessments such as the Glasgow Coma Scale or Richmond Agitation-Sedation Scale). The ER model also contains other standard inpatient EDW tables (e.g. vitals, labs) and can be implemented as Structured Query Language (SQL) views or in other database structures, as CLIF is language agnostic.

### Minimum Common ICU Data Elements and Preservation of source EHR Data

The National Institutes of Health (NIH) defines a Common Data Elements (CDE) as a “standardized, precisely defined question, paired with a set of allowable responses, used systematically across different sites, studies, or clinical trials to ensure consistent data collection.”^16^ For each CLIF table, we developed a minimum set of Common ICU Data Elements (mCIDE) denoted with the “_category” suffix. Each CIDE 1) represents a precisely defined clinical entity relevant to critical illness and 2) has a limited set of permissible values. Whenever possible, we adopted NIH-endorsed CDEs into CLIF.^17^ We created several novel CIDEs for CLIF, such as modes of mechanical ventilation (mode_category). Recognizing that our mCIDE is insufficient for all research purposes, we preserve source EHR data elements in “_name” fields.” For example, lab_name (e.g., “UCM_LAB HEMOGLOBIN - AUTOMATED”) preserves the specific lab test name as used at the site, while lab_category (e.g., “Hemoglobin”) maps this test to a specific permissible value of the lab_category CDE. This structure creates standardized ICU elements while preserving original data labels for quality control.

### CLIF open-source commitment, AI disclosure, and IRB approval

CLIF continues to mature via our collaborative development process supported by git version control and is released under the Apache 2.0 license to ensure open access and broad usage rights. Our consortium website and code repository (clif-consortium.github.io/website/) contains data dictionaries, ETL pipeline examples, quality control scripts, and complete analysis code for each case study. Each of the eight CLIF consortium sites independently received IRB approval to conduct observational studies or to build and/or quality-check a research EDW (see Supplement Table E1 for IRB details). No patient-level data was shared between sites at any point. We used AI-assisted technologies, including large language models (LLMs), to edit the manuscript and code analysis scripts. The authors carefully reviewed and verified all AI-generated content to ensure accuracy and originality. All quoted material is properly cited.

### Cohort discovery and federated analytics in the CLIF consortium

We conducted two proof-of-concept case studies: (1) development and external validation of a novel multivariable AI ICU mortality prediction model and (2) external validation of a previously developed subphenotyping algorithm for temperature trajectories in critical illness.^18^ We used the same cohort-discovery script and the CLIF admission-discharge-transfer (ADT) and patient tables to identify all adults (≥18 years) admitted to an ICU within 48 hours of hospitalization and staying at least 24 hours, from January 1, 2020, to December 31, 2021, at each site across the consortium (Figure E1). We chose these inclusion criteria to identify the general ICU population, excluding patients who die shortly after ICU admission or are admitted to the ICU for non-critical reasons (e.g., to facilitate a procedure). Using standardized outlier handling scripts and consortium-defined outlier ranges, we removed clear data entry errors (e.g., a heart rate of 1,000 beats per minute).

### Case Study I: Development and External Validation of an In-Hospital Mortality Model for ICU Patients

Accurate and reliable hospital mortality predictions for critically ill patients may help clinical teams prioritize therapeutic interventions, facilitate more informed shared decision-making around goals of care, and optimize resource allocation within healthcare systems. Existing prediction models are limited by suboptimal accuracy and significant performance variation across hospitals and differential performance among vulnerable populations may exacerbate baseline inequities in access to (and quality of) critical care. ^19,20–23^ In this case study, we developed and externally validated an AI model to predict hospital mortality using clinical data from the first 24 hours in the ICU.

We trained a light gradient boosted machine binary classifier (LightGBM)^24^ to predict in-hospital death on separate cohort of ICU admissions in CLIF format from Rush University Medical Center using data from 2019, 2022, and 2023, performing hyperparameter tuning through a grid search with 5-fold cross validation. We selected LightGBM for its high discrimination and its ability to handle missing data without the need for imputation or exclusion of cases with high levels of missingness.^24^ We selected 30 candidate predictors *a priori* from hours 0–24 in the ICU (Table E2) based on literature review, our clinical experience, and expected low levels of missingness. We then saved the prediction model object in python and the general LGBM TXT format to the shared publicly available consortium repository.

We then evaluated this model on the 2020–2021 cohort described above at Rush and all other CLIF sites using a federated approach with a common model evaluation script, the model object, and each site’s local CLIF database. To comprehensively assess the model’s generalizability, we applied the TRIPOD-AI checklist across all test sites.^25^ Our evaluation focused on three key aspects: discrimination using the area under the receiver operating characteristic curve (AUC), calibration using Brier scores and calibration plots, and clinical utility through decision curve analysis.^25,26^

### Case Study II: Temperature Trajectory Subphenotyping

Growing recognition of heterogeneity within critical illness syndromes has led to the emergence of algorithmic clinical subphenotyping as a means of generating new hypotheses for investigation, improving clinical prognostication, and characterizing heterogeneous treatment effects.^27^ Despite the potential value of these advances in precision medicine, subphenotyping models are rarely externally validated.^28^

In our second case study, we externally validated a previously-developed unsupervised model to subphenotype infected patients in the hospital according to longitudinal temperature trajectories.^18^ This approach uses group-based trajectory modeling and patient temperature trends over 72 hours to assign patient encounters into one of four mutually-exclusive subphenotypes: normothermic (NT), hypothermic (HT), hyperthermic fast-resolver (HFR), and hyperthermic slow-resolver (HSR). In 1- and 2-hospital studies of patients with undifferentiated suspected infection and COVID-19 (regardless of ICU status), these subphenotypes have demonstrated distinct immune and inflammatory profiles and differential outcomes, including ICU utilization and mortality.^29–31^ However, the temperature trajectory model has not previously been evaluated within a broader critically ill population.

We developed analysis scripts that standardized body temperature measurements during the first 72 hours of ICU admission and classified each patient into the temperature trajectory subgroup with the lowest sum of the mean squared errors between the patient’s observed temperature and the subphenotype’s reference trajectory. Finally, we assessed differences in patient characteristics by subphenotype and the association of subphenotypes with in-hospital mortality and receipt of invasive mechanical ventilation using multivariable logistic regression adjusted for patient age, sex, race, and ethnicity.

## RESULTS:

To date, we have established CLIF databases at eight US health systems, comprising 33 unique hospitals and 94,356 ICU admissions (Figure E2, Table 2). Health system-level populations were similar in terms of age (mean 60.6 years overall [standard deviation 17.2], range 56.4 [18.5] to 63.2 [17.5]) and sex (45% female overall, range 41–47% female). As expected, each site’s population differed substantially by race, ranging from 7% to 66% Black. Clinical outcomes differed moderately across systems. Overall, patients received invasive mechanical ventilation in 35,789 encounters (38% overall, range 28–50%), and 8,920 patients died in the hospital (9.5% overall, range 7.4–13%).

### Development and External Validation of an In-Hospital Mortality Model for ICU Patients

The separate Rush training cohort consisted of 17,139 ICU admissions with similar demographics to the Rush test cohort (Table E3). The final model hyperparameters of the lightGBM are described in the Supplement (Table E4). The most important features in the model were: minimum albumin level, maximum aspartate aminotransferase (AST), minimum pulse rate, minimum diastolic blood pressure (DBP), and mean aspartate aminotransferase (AST) (see variable importance plot Figure E3).

**Figure 2.**
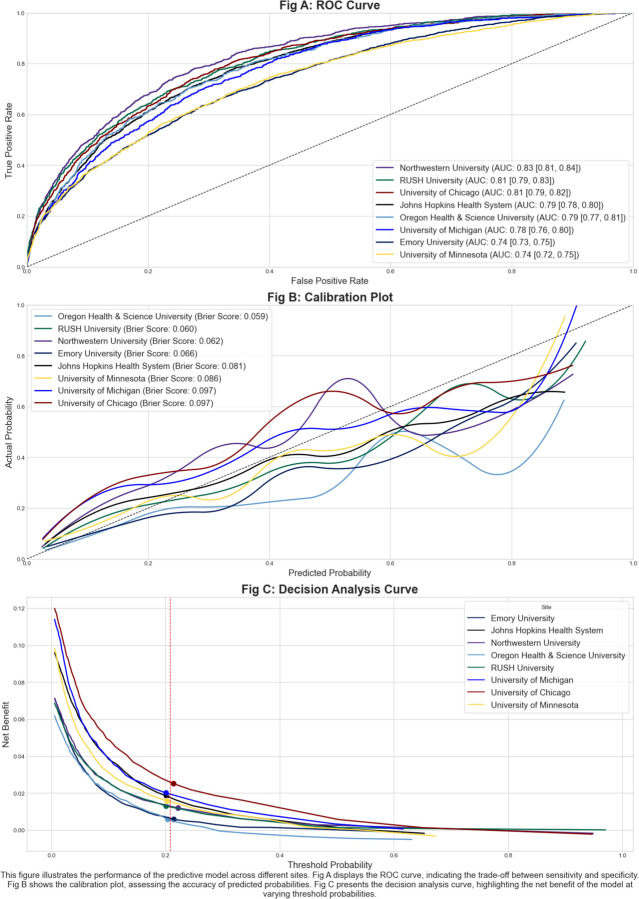
Mortality model performance validation via receiver-operator characteristic curves (A), calibration curves (B), and decision curves (C).

In the hold-out test cohort of 94,356 ICU admissions (Table 1), the AUC for predicting in-hospital mortality varied across sites, ranging from 0.74 to 0.81. Specifically, the Chicagoland health systems of Rush, Northwestern, and University of Chicago exhibited the highest AUCs, with values of 0.81 [95% CI: 0.79–0.83], 0.83 [0.81 – 0.84], and 0.81 [95% CI: 0.79–0.82], respectively. In contrast, Emory and the University of Minnesota reported the lowest AUCs, both at 0.74 [95% CI: 0.73–0.75] and 0.74 [95% CI: 0.72–0.75]. These performance metrics are summarized in Figure 2a. Calibration was assessed using Brier scores, which ranged from 0.059 at Oregon Health & Science University (OHSU) (indicating the best calibration) to 0.097 at the University of Michigan and the University of Chicago. The calibration plot (Figure 2b) demonstrates predicted versus observed probabilities across all sites, with most curves closely following the diagonal line at lower probabilities but some deviation at higher probabilities. Net benefit is a weighted average of true positives and false positives intended to quantify the clinical utility of a model at different treatment thresholds varied across sites, as shown in the decision-curve analysis in Figure 2c.^26^ At the high-risk threshold of a 0.3 probability of death determined *a priori* by Rush University, the model conferred the highest net benefit to the University of Chicago (0.025), followed by the University of Michigan (0.020), and John Hopkins (0.018). The model lead to the lowest net benefits at Emory and Oregon Health & Science University both at 0.006. The model had positive net benefit at all sites at the threshold, indicating it increased utility compared to a “treat none” strategy (net benefit = 0 by definition) and a “treat all” strategy (net benefit ranging from −0.24 to −0.32).

### Temperature Trajectory Subphenotypes

**Figure 3.**
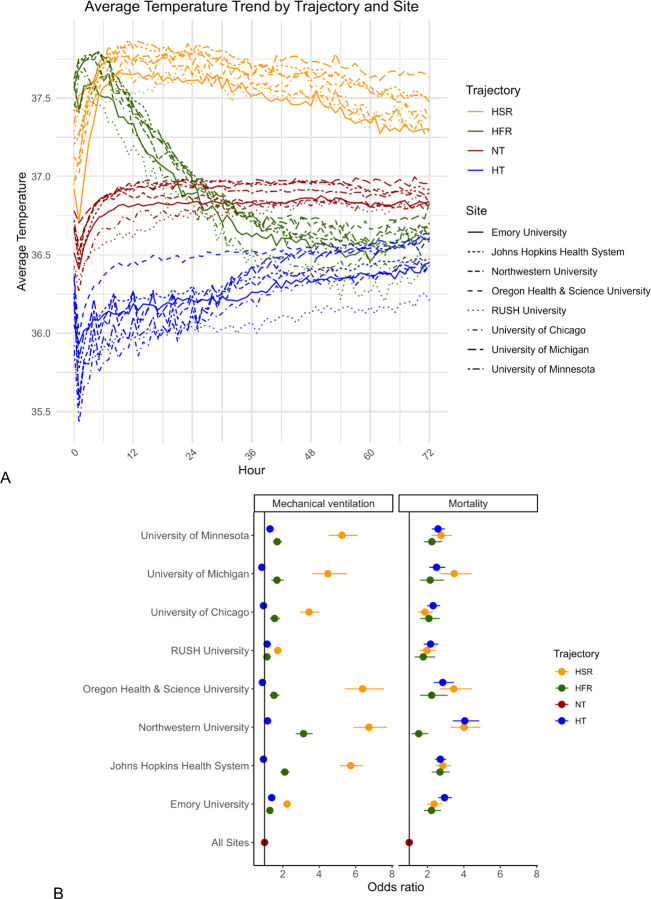
Temperature trends (A) and adjusted odds for ICU outcomes (B) across temperature trajectory subphenotypes at all sites.

Across the eight participating institutions, this case study analyzed 94,290 ICU admissions, excluding 66 (0.07%) ICU admissions without any recorded temperatures (Table E4). Each subphenotype had a consistent observed temperature trajectory across all sites (Figure 3A). Normothermic encounters had the highest prevalence overall and at each site (range 43–68%), followed by hypothermic encounters (range 13–34%), hyperthermic slow resolvers (range 7.8–16%), and hyperthermic fast resolvers (range 6.2–7.9%). The distribution of patient characteristics across subphenotypes was similar across sites. Hypothermic patients were consistently older than other groups (range 61.4–68.9) and hyperthermic slow resolvers were the youngest group at all sites but one (range 47.8–60.0).

Several consistent outcome patterns were observed across sites. Hyperthermic slow resolvers had the highest rates of invasive mechanical ventilation at all sites (range 38–79%). Mechanical ventilation rates were lowest among normothermic and hypothermic patients. Mortality was lowest among normothermic patients (range 3.8–8.2%) and highest among hypothermic (9.9–18.2%) and hyperthermic slow resolving (8.5–20.3%) patients.

After adjustment for age, sex, race, and ethnicity, subphenotype membership was consistently and independently associated with these outcomes (Figure 3B). HSR and HFR subphenotypes were associated with significantly increased odds for invasive mechanical ventilation (IMV) (as compared to normothermic) at all sites. Additionally, HSR, HFR, and HT subphenotypes were associated with significantly increased odds for mortality at all sites.

## DISCUSSION

We developed CLIF to standardize complex ICU data into a consistent, longitudinal format necessary for transparent and reproducible critical care research. In two proof-of-concept studies involving nearly 100,000 diverse critically ill patients, we demonstrated the potential of CLIF and a federated consortium research approach.

In our first case study, the mortality model demonstrated good discrimination and calibration in the internal RUSH validation cohort. However, its performance varied across seven other CLIF consortium sites. Decision curve analysis revealed positive but varying clinical utility across sites, highlighting the model’s sensitivity to local clinical and operational differences. For example, significant calibration slope errors (overestimating mortality) in the OHSU and Emory test sets led to lower net benefit and less clinical utility at the specified clinical decision threshold. These findings underscore the challenge of generalizing prognostic models across diverse healthcare settings in a one-size-fits-all fashion.^19^ This case study demonstrates CLIF’s value in facilitating rigorous, multi-site evaluations of predictive models. The natural next step is the development and validation of a set of generalizable ICU prediction models trained across the entire CLIF consortium using decentralized federated learning.^32^

Our second case study expands Bhavani et al.’s temperature trajectory subphenotyping model to a larger, broader, and more diverse cohort using the CLIF framework.^18^ While Bhavani et al. identified four subphenotypes in a sepsis-specific cohort—hyperthermic slow resolvers, hyperthermic fast resolvers, normothermic, and hypothermic—we applied this model to undifferentiated patients with critical illness.^18^ Mirroring prior findings in sepsis, we observed the highest mortality rates in the hypothermic group, suggesting this subphenotype robustly predicts outcomes in a general critical illness population. The consistent association of temperature trajectories with mortality and mechanical ventilation across health systems highlights the potential of longitudinal data analysis for critical illness phenotyping and personalizing ICU treatment.^33^

### Limitations and areas for improvement

The CLIF format has limitations and we are actively seeking feedback on the project. First, CLIF required substantial data science and critical care expertise to implement at each consortium site. The consortium is developing open-source tools to help diverse healthcare institutions adopt CLIF in an automated, scalable, and flexible manner. These ETL tools must be sensitive to the evolving landscape of healthcare data storage as institutions shift from traditional warehouses to flexible, scalable data lakes. This change allows CLIF to play a key role in improving healthcare data representation. CLIF helps transform often hard-to-interpret raw data in the ICU into more valuable, integrated information ready for advanced analysis. Second, CLIF is currently not linked to established interoperability standards like Health Level Seven (HL7) Fast Healthcare Interoperability Resources (FHIR). Future data engineering directions include the development of HL7 FHIR queries to generate real-time CLIF tables.

Third, the case studies presented in this manuscript used ADT data to define the critical illness population. This approach is susceptible to selection bias and left-censoring. However, a key strength of the CLIF framework is it is designed to represent critical illness wherever it occurs in the hospital.^34^ This means that future CLIF analyses do not have to rely on a patient’s physical location or ADT data to define a disease state. Fourth, while CLIF’s diverse data elements are not a substitute for proper longitudinal study design. Limitations like incomplete data or collider bias can still confound CLIF studies, especially when hospitalization patterns vary among vulnerable groups.^35^ Fortunately, advances in causal inference, such as target trial emulation, provide a clear roadmap for the CLIF consortium.

Finally, while our work demonstrates the benefits of federated analysis, ideally, we would release de-identified versions of our CLIF databases for public use. Once CLIF’s utility as a format is firmly established, we hope to make the case to our health systems leadership to make the large investment required to follow the inspirational example of MIMIC.^36^

### Conclusions and future directions

We developed and implemented a Common Longitudinal ICU data Format (CLIF) across 8 diverse health systems and demonstrated its value in two proof-of-concept case studies. We believe our open-source approach will make CLIF a broadly appealing target format for representing critical illness. Aspirational future directions for CLIF include 1) data format for pragmatic EHR-based trials, 2) rigorous target trial emulation framework for causal inference, 3) real-time critical care quality dashboards, and 4) foundational multi-modal AI models of critical illness.

## Supplementary Material

Supplement 1

## Figures and Tables

**Table 1.** Practical Challenges to EHR Data Science in the Hospital

Challenge	Description	Example
**Complex longitudinal data with differing frequencies drawn from multiple sources**	Diverse domains such as vital signs, laboratory measurements, medications, and respiratory support require different data structures for representation and analysis.	Vital signs are frequently recorded during hospitalization (e.g. hourly) while laboratory results occur much less frequently and can be distributed across different record id numbers for the same patient (e.g., tests obtained in a clinic before the patient is referred to the emergency department or admitted). Microbiology tests are similarly recorded but are further complicated by the possibility of multiple observations per test (e.g., blood culture positive for multiple organisms) and nested antimicrobial susceptibility testing.
**Interdependent data**	Complex care processes are implicitly embedded in the presence, absence, frequency, and content of structured data.	Continuous neuromuscular blockade (recorded in the medication administration table) requires invasive mechanical ventilation (recorded in the respiratory flowsheet tables).
**Temporally-dependent data**		Sepsis onset is defined by complex temporal heuristics involving the sequencing and timing of antimicrobials, infectious tests, and abnormal physiology.
**Inefficient and inaccurate data capture**	Many bedside measurements (e.g., vital signs, respiratory parameters) require manual recording or human validation of automatically recorded data before they are available in the EHR.	Respiratory flowsheets often contain carryforward and copy/paste observations, leading to internal inconsistencies (e.g., patients recorded as receiving low-flow nasal oxygen and invasive ventilation simultaneously).
**Complex data storage**	ICU data storage is fragmented across different systems (e.g., ventilator data, laboratory systems, vital signs), making comprehensive data analysis challenging without sophisticated integration efforts. Diverse end-user needs and goals (e.g., operational quality reporting vs. clinical research) lead enterprise and research data warehouses to adopt a “one size fits all” content and format approach for EHR data, which may not be optimal for specific research or operational needs.	ICU data on ventilator settings may be stored separately from laboratory results or vital signs, requiring complex data integration for analysis. Additionally, the “one size fits all” approach in data warehouses can result in data formats that are not ideal for specific research tasks, such as temporal analyses or patient- specific interventions.
**Local idiosyncrasies**	ICU practices and data recording can vary significantly between institutions, with local protocols influencing how data is recorded and stored, leading to variability that complicates multicenter studies.	ICU triage decisions, such as when to escalate care to invasive ventilation, are often based on local protocols, which can differ significantly between hospitals, leading to challenges in generalizing study findings across different settings.

**Table 2.** Characteristics and outcomes of ICU patient encounters in 2020–2021 across CLIF sites.

Site	Emory University	JHHS	University of Minnesota	University of Michigan	Northwestern Medicine	OHSU	Rush University	University of Chicago	CLIF Consortium (Combined)
**Encounters, n**	19,923	18,325	11,576	6,877	10,625*	9,046	9,905	8,079	94,356
**Hospitals, n**	4	5	11	1	8	2	1	1	33
**Age (years), mean (SD)**	61.2 (16.5)	60.6 (17.4)	61.8 (17.3)	59.0 (16.2)	63.2 (17.5)	59.9 (17.3)	59.6 (16.9)	56.4 (18.5)	60.6 (17.2)
**Female n (%)**	9406 (47%)	8428 (46%)	5128 (44%)	2834 (41%)	4840 (46%)	3814 (42%)	4687 (47%)	3422 (42%)	42559 (45%)
**Race n (%)**									
**Asian**	671 (3.4%)	765 (4.2%)	756 (6.5%)	135 (2.0%)	352 (3.3%)	276 (3.0%)	329 (3.3%)	150 (1.9%)	3434 (3.6%)
**Black**	9019 (45%)	5881 (32%)	824 (7.1%)	826 (12%)	1272 (12.0%)	775 (8.6%)	4012 (41%)	5307 (66%)	27916 (30%)
**White**	9048 (45%)	10128 (55%)	9503 (82%)	5429 (79%)	8005 (75.3%)	7525 (83%)	3717 (38%)	2015 (25%)	55370 (59%)
**Others**	1185 (5.9%)	1551 (8.5%)	493 (4.3%)	487 (7.1%)	996 (9.4%)	470 (5.2%)	1847 (19%)	607 (7.5%)	7636 (8%)
**Ethnicity n (%)**									
**Hispanic or Latino**	761 (3.8%)	1208 (6.6%)	224 (1.9%)	201 (2.9%)	1004 (9.4%)	788 (8.7%)	1983 (20%)	497 (6.2%)	6666 (7.0%)
**Not Hispanic**	19162 (96%)	17117 (93%)	11352 (98%)	6676 (97%)	9851 (90.2%)	8258 (91%)	7922 (80%)	7582 (94%)	87920 (93%)
**Mechanical Ventilation, n (%)**	9852 (50%)	6595 (36%)	4059 (35%)	3358 (49%)	3034 (29%)	2563 (28%)	2862 (29%)	3466 (49%)	35789 (38%)
**Hospital mortality, n (%)**	1529 (7.7%)	1851 (10%)	1193 (10%)	817 (12%)	809 (7.9%)	983 (11%)	730 (7.4%)	1008 (13%)	8920 (9.5%)

OHSU, Oregon Health & Science University; JHHS, The Johns Hopkins Health System Corporation CLIF, Common Longitudinal ICU data Format; SD, standard deviation *Subset of approximately 50% of Northwestern ICU encounters; only encounters that had vitals data currently available in the NU-CRITICAL database^37^ were included in this analysis
