# Supplementary material for: A Common Longitudinal Intensive Care Unit data Format (CLIF) to enable multi-institutional federated critical illness research
Source: medRxiv. 2024 Sep 4:2024.09.04.24313058. Preprint. [Version 1] doi: 10.1101/2024.09.04.24313058 (PMC11398431; doi:10.1101/2024.09.04.24313058)

Supplementary Tables and Figures:

Title: A Common Longitudinal Intensive Care Unit data Format (CLIF) to enable multi-institutional federated critical illness research

Running Title: CLIF: Standardizing ICU Data for Federated Research

Table E1: IRB Details for CLIF Consortium Sites

This supplement provides IRB approval numbers for observational studies of critically ill patients and EDW building/quality-checking activities conducted at CLIF consortium sites.

CLIF Consortium Site	IRB Number
Oregon Health & Science University	00025188
University of Minnesota	STUDY00014815
University of Michigan	HUM00144238
Northwestern Medicine	STU00202840
University of Chicago	IRB20-1823
Rush University	20082408-IRB01
The Johns Hopkins Health System Corporation	IRB00421735
Emory University	Study 1815

Table E2: Features of the Machine Learning Model Predicting ICU Mortality During the First 24 Hours

Category	Feature	Description
**Demographics**	isfemale	Gender (1 for female, 0 for male)
	age	Age of the patient at encounter
**Vital Signs**	min_pulse	Minimum pulse rate
	max_pulse	Maximum pulse rate
	avg_pulse	Average pulse rate
	min_sbp	Minimum systolic blood pressure
	max_sbp	Maximum systolic blood pressure
	avg_sbp	Average systolic blood pressure
	min_dbp	Minimum diastolic blood pressure
	max_dbp	Maximum diastolic blood pressure
	avg_dbp	Average diastolic blood pressure
	min_temp_c	Minimum temperature in °C
	max_temp_c	Maximum temperature in °C
	avg_temp_c	Average temperature in °C
**Body Mass Index & Weight**	min_bmi	Minimum BMI
	max_bmi	Maximum BMI
	avg_bmi	Average BMI
	min_weight_kg	Minimum weight in kg
	max_weight_kg	Maximum weight in kg
	avg_weight_kg	Average weight in kg
**Laboratory Results**	albumin_min	Minimum albumin level
	albumin_max	Maximum albumin level
	albumin_mean	Mean albumin level
	alkaline_phosphatase_min	Minimum alkaline phosphatase level
	alkaline_phosphatase_max	Maximum alkaline phosphatase level
	alkaline_phosphatase_mean	Mean alkaline phosphatase level
	ast_min	Minimum AST (aspartate aminotransferase) level
	ast_max	Maximum AST (aspartate aminotransferase) level
	ast_mean	Mean AST (aspartate aminotransferase) level
	bilirubin_conjugated_min	Minimum conjugated bilirubin level
	bilirubin_conjugated_max	Maximum conjugated bilirubin level

	bilirubin_conjugated_mean	Mean conjugated bilirubin level
	bilirubin_total_min	Minimum total bilirubin level
	bilirubin_total_max	Maximum total bilirubin level
	bilirubin_total_mean	Mean total bilirubin level
	calcium_min	Minimum calcium level
	calcium_max	Maximum calcium level
	calcium_mean	Mean calcium level
	chloride_min	Minimum chloride level
	chloride_max	Maximum chloride level
	chloride_mean	Mean chloride level
	glucose_serum_min	Minimum serum glucose level
	glucose_serum_max	Maximum serum glucose level
	glucose_serum_mean	Mean serum glucose level
	hemoglobin_min	Minimum hemoglobin level
	hemoglobin_max	Maximum hemoglobin level
	hemoglobin_mean	Mean hemoglobin level
	lymphocyte_min	Minimum lymphocyte count
	lymphocyte_max	Maximum lymphocyte count
	lymphocyte_mean	Mean lymphocyte count
	monocyte_min	Minimum monocyte count
	monocyte_max	Maximum monocyte count
	monocyte_mean	Mean monocyte count
	neutrophil_min	Minimum neutrophil count
	neutrophil_max	Maximum neutrophil count
	neutrophil_mean	Mean neutrophil count
	platelet count_min	Minimum platelet count
	platelet count_max	Maximum platelet count
	platelet count_mean	Mean platelet count
	potassium_min	Minimum potassium level
	potassium_max	Maximum potassium level
	potassium_mean	Mean potassium level
	sodium_min	Minimum sodium level
	sodium_max	Maximum sodium level
	sodium_mean	Mean sodium level
	total_protein_min	Minimum total protein level
	total_protein_max	Maximum total protein level
	total_protein_mean	Mean total protein level
	wbc_min	Minimum white blood cell count
	wbc_max	Maximum white blood cell count
	wbc_mean	Mean white blood cell count

Table E3: Derivation Cohort Characteristics for Inpatient Mortality Model at Rush University

Site	RUSH
Encounters, n	17,139
Hospitals, n	1
Age (years), mean (SD)	60.3 (17.0)
Female n (%)	8140 (47.5%)
Race n (%)	
Asian	543 (3.2%)
Black	6458 (37.7%)
White	6932 (40.4%)
Others	3206 (18.7%)
Ethnicity n (%)	
Hispanic or Latino	3135 (18.3%)
Not Hispanic	14004 (81.7%)
Mechanical Ventilation, n (%)	4495 (26.2%)
Hospital mortality, n (%)	1059 (6.2%)

Table E4: Hyperparameters for Inpatient Mortality LightGBM Model Derived at Rush

**Parameter**	**Value**
boosting	gbdt
objective	binary
metric	['binary_logloss']
tree_learner	serial
device_type	gpu
data_sample_strategy	bagging
num_iterations	50
learning_rate	0.1
num_leaves	31
num_threads	4
seed	0
deterministic	FALSE
force_col_wise	TRUE
force_row_wise	FALSE
histogram_pool_size	-1
max_depth	10
min_data_in_leaf	20

min_sum_hessian_in_leaf	0.001
bagging_fraction	1
pos_bagging_fraction	1
neg_bagging_fraction	1
bagging_freq	0
bagging_seed	3
feature_fraction	1
feature_fraction_bynode	1
feature_fraction_seed	2
extra_trees	FALSE
extra_seed	6
early_stopping_round	0
first_metric_only	FALSE
max_delta_step	0
lambda_l1	0
lambda_l2	0
linear_lambda	0
min_gain_to_split	0
drop_rate	0.1

max_drop	50
skip_drop	0.5
xgboost_dart_mode	FALSE
uniform_drop	FALSE
drop_seed	4
top_rate	0.2
other_rate	0.1
min_data_per_group	100
max_cat_threshold	32
cat_l2	10
cat_smooth	10
max_cat_to_onehot	4
top_k	20
monotone_constraints_method	basic
monotone_penalty	0
refit_decay_rate	0.9
cegb_tradeoff	1
cegb_penalty_split	0
path_smooth	0

verbosity	1
saved_feature_importance_type	0
use_quantized_grad	FALSE
num_grad_quant_bins	4
quant_train_renew_leaf	FALSE
stochastic_rounding	TRUE
linear_tree	FALSE
max_bin	255
min_data_in_bin	3
bin_construct_sample_cnt	200000
data_random_seed	1
is_enable_sparse	TRUE
enable_bundle	TRUE
use_missing	TRUE
zero_as_missing	FALSE
feature_pre_filter	TRUE
pre_partition	FALSE
two_round	FALSE
header	FALSE

precise_float_parser	FALSE
objective_seed	5
num_class	1
is_unbalance	FALSE
scale_pos_weight	1
sigmoid	1
boost_from_average	TRUE
reg_sqrt	FALSE
alpha	0.9
fair_c	1
poisson_max_delta_step	0.7
tweedie_variance_power	1.5
lambdarank_truncation_level	30
lambdarank_norm	TRUE
lambdarank_position_bias_regularization	0
multi_error_top_k	1
num_machines	1
local_listen_port	12400
time_out	120

gpu_platform_id	-1
gpu_device_id	-1
gpu_use_dp	FALSE
num_gpu	1

Table E5. Temperature Trajectory Data by CLIF Site

Institution	_	HSR	HFR	Normothermic	Hypothermic	P-value
Emory University	Encounters, n	2441	1487	11186	4805	-
	Age (years), mean (SD)	56.7 (15.9)	58.7 (16.7)	61.5 (16.4)	63.7 (16.4)	<0.001
	Male n (%)	1496 (61.3%)	809 (54.4%)	5772 (51.6%)	2437 (50.7%)	<0.001
	Mechanical Ventilation, n (%)	1568 (64.2%)	756 (50.8%)	4923 (44%)	2603 (54.2%)	<0.001
	Hospital mortality, n (%)	233 (9.5%)	134 (9%)	521 (4.7%)	640 (13.3%)	<0.001
Johns Hopkins Health System Corporation	Encounters, n	1869	1143	10896	4417	-
	Age (years), mean (SD)	55.5 (17.6)	58.4 (18.2)	60.6 (17.2)	63.3 (16.9)	<0.001
	Male n (%)	1172 (62.7%)	610 (53.4%)	5822 (53.4%)	2293 (51.9%)	<0.001
	Mechanical Ventilation, n (%)	1350 (72.2%)	554 (48.5%)	3371 (30.9%)	1320 (29.9%)	<0.001
	Hospital mortality, n (%)	268 (14.3%)	167 (14.6%)	697 (6.4%)	719 (16.3%)	<0.001
Northwestern University	Encounters, n	1106	834	7262	1423	-
	Age (years), mean (SD)	60 (17)	61.6 (17.1)	62.8 (17.5)	68.9 (16.5)	<0.001
	Male n (%)	661 (59.8%)	504 (60.4%)	3889 (53.6%)	731 (51.4%)	<0.001
	Mechanical Ventilation, n (%)	723 (65.4%)	393 (47.1%)	1575 (21.7%)	343 (24.1%)	<0.001
	Hospital mortality, n (%)	167 (15.1)	55(6.6)	330 (4.5)	257 (18.1)	<0.001
Oregon Health & Science University	Encounters, n	871	561	5154	2460	-
	Age (years), mean (SD)	54.8 (17.3)	56.4 (17.8)	59 (17.1)	64.6 (16.4)	<0.001
	Male n (%)	552 (63.4%)	334 (59.5%)	2936 (57%)	1410 (57.3%)	0.004
	Mechanical Ventilation, n (%)	667 (76.6%)	244 (43.5%)	1693 (32.8%)	712 (28.9%)	<0.001
	Hospital mortality, n (%)	102 (11.7%)	45 (8%)	197 (3.8%)	263 (10.7%)	<0.001
Rush	Encounters, n	1562	707	4291	3328	-

**University**	**Age (years), mean (SD)**	55.6 (17)	55.8 (17.5)	59.3 (16.8)	62.6 (16.2)	<0.001
	**Male n (%)**	902 (57.7%)	376 (53.2%)	2234 (52.1%)	1699 (51.1%)	<0.001
	**Mechanical Ventilation, n (%)**	597 (38.2%)	203 (28.7%)	1109 (25.8%)	942 (28.3%)	<0.001
	**Hospital mortality, n (%)**	133 (8.5%)	55 (7.8%)	198 (4.6%)	330 (9.9%)	<0.001
**University of Chicago**	**Encounters, n**	982	564	3982	2522	-
	**Age (years), mean (SD)**	47.7 (18.2)	52.1 (18.4)	55.6 (18.3)	61.5 (17.3)	<0.001
	**Male n (%)**	690 (70.3%)	327 (58%)	2274 (57.1%)	1344 (53.3%)	<0.001
	**Mechanical Ventilation, n (%)**	682 (69.5%)	281 (49.8%)	1551 (39%)	937 (37.2)	<0.001
	**Hospital mortality, n (%)**	130 (13.2%)	85 (15.1%)	85 (15.1%)	464 (18.4)	<0.001
**University of Michigan**	**Encounters, n**	590	449	3955	1867	-
	**Age (years), mean (SD)**	53.8 (17.5)	57.7 (15.8)	58.2 (16)	62.6 (15.4)	<0.001
	**Male n (%)**	359 (60.8%)	279 (62.1%)	2272 (57.4%)	1128 (60.4%)	0.04
	**Mechanical Ventilation, n (%)**	467 (79.2%)	267 (59.5%)	1825 (46.1%)	793 (42.5%)	<0.001
	**Hospital mortality, n (%)**	120 (20.3%)	65 (14.5%)	289 (7.3%)	330 (17.7%)	<0.001
**University of Minnesota**	**Encounters, n**	908	820	7355	2493	-
	**Age (years), mean (SD)**	58.4 (17.3)	60.9 (17.5)	61 (17.4)	65.7 (16.6)	<0.001
	**Male n (%)**	553 (60.9%)	454 (55.4%)	4038 (54.9%)	1403 (56.3%)	0.007
	**Mechanical Ventilation, n (%)**	634 (69.8%)	344 (42%)	2211 (30.1%)	870 (34.9%)	<0.001
	**Hospital mortality, n (%)**	144 (15.9%)	114 (13.9%)	504 (6.9%)	431 (17.3%)	<0.001

Abbreviations: (HSR) = hyperthermic, slow resolvers; (HFR)= hyperthermic, fast resolvers

Supplement Figure Legend:

Figure E1: Inclusion and Exclusion Criteria for ICU CLIF Case Studies
This figure illustrates the detailed criteria used for including and excluding cases in the ICU CLIF case studies.

Figure E2: US States Represented by CLIF
This map highlights the US states represented in the CLIF study, with participating institutions listed alongside their respective states. The institutions include:
Oregon Health & Science University (Oregon)
University of Minnesota (Minnesota)
University of Michigan (Michigan)
Northwestern Medicine (Illinois)
UChicago Medicine (Illinois)
Rush University (Illinois)
Johns Hopkins Medicine (Maryland)
Emory University (Georgia)

Figure E3: Variable Importance Plot for Inpatient Mortality Prediction Model
Feature Importance (Gain): This plot ranks the features (variables) in the model based on their significance in predicting inpatient mortality. The importance of each feature is measured using a metric called "Gain," which reflects how much each feature improves the model's accuracy in predicting the likelihood of a patient's survival during hospitalization.
Gain: The higher the Gain for a feature, the more it contributes to the model's ability to predict the target outcome, which in this case is inpatient mortality. Gain measures the impact of each feature on the decision-making process of the model, with higher Gain values indicating a greater influence on predicting whether a patient will survive or not.
For example, if "Minimum Albumin" has the highest Gain value in the plot, it means that this feature is the most important for the model's predictions of inpatient mortality. This implies that lower levels of albuminare strongly associated with a higher risk of mortality in hospitalized patients, more so than other measured variables.

Legend:
WBC: White Blood Cell count
AST: Aspartate Aminotransferase, an enzyme found in the liver and other tissues
BP: Blood Pressure

Figure E1: Inclusion and Exclusion Criteria for ICU CLIF Case Studies

Inclusion Criteria

- ICU admission $\leq$ 48h after hospital admission
- ICU stay $\geq$ 24 hours
- Encounter between Jan 1, 2020 - Dec 31, 2021
- Age $\geq$ 18 years at admission
- Gender information available
- Disposition recorded (survived or died during hospitalization)

Exclusion Criteria

- ICU admission $>$ 48h after hospital admission
- ICU stay $<$ 24 hours
- Encounter outside Jan 1, 2020 - Dec 31, 2021
- Age $<$ 18 years at admission
- Gender information unavailable
- Disposition not recorded (unknown if survived or died during hospitalization)

Figure E2. US States represented by CLIF

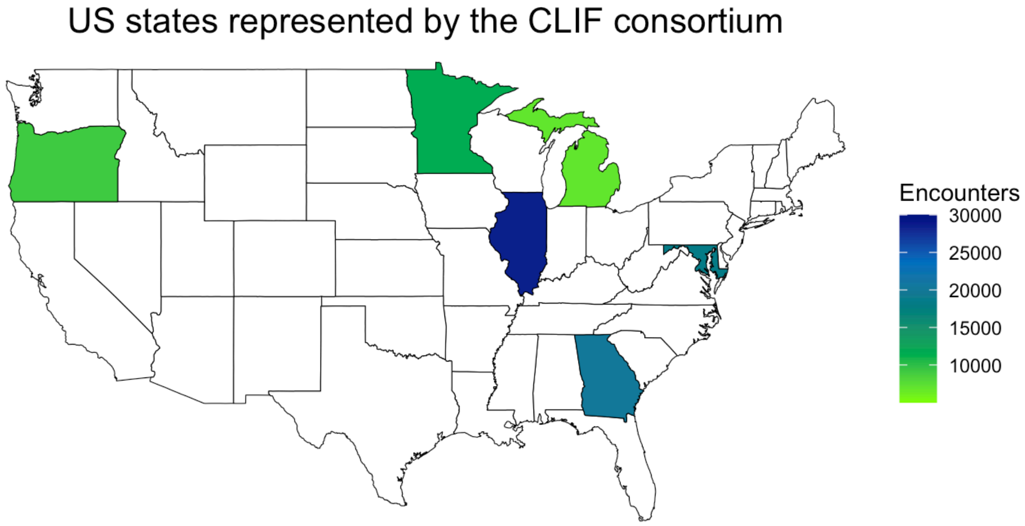

Figure E3: Variable Importance Plot for Inpatient Mortality Prediction Model

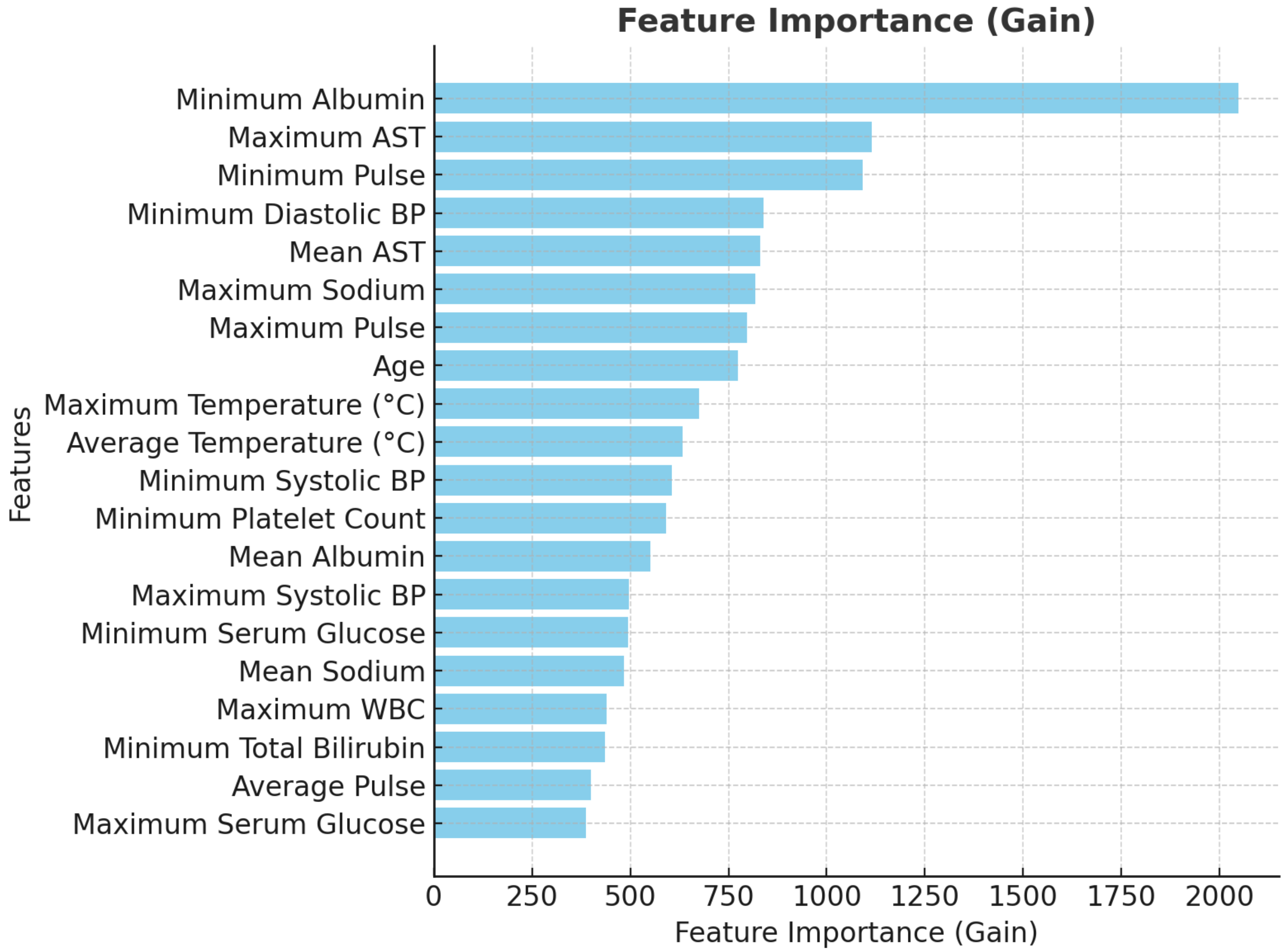

Supplement: Supplement 1 [file media-1.pdf]
